# Treatment of Ovarian Clear Cell Carcinoma: A Case of Successful Management With Targeted Therapies

**DOI:** 10.1155/crog/1319978

**Published:** 2025-07-12

**Authors:** Sydney Pence, Kellie Rath, Aine Clements

**Affiliations:** ^1^Department of Obstetrics and Gynecology, Ohio Health-Riverside Methodist Hospital, Columbus, Ohio, USA; ^2^Department of Gynecological Oncology, Ohio Health-Riverside Methodist Hospital, Columbus, Ohio, USA

**Keywords:** ipilimumab, nivolumab, ovarian clear cell carcinoma

## Abstract

**Background:** Advanced stages of ovarian clear cell carcinoma have poorer prognoses than many other ovarian cancers. When standard treatments are ineffective, alternative options are needed.

**Case:** A 56-year-old woman was diagnosed with ovarian clear cell carcinoma and treated with surgical management as well as carboplatin and paclitaxel. At recurrence, her disease progressed despite multiple chemotherapy regimens. A durable response was achieved first with alpelisib, chosen based on genomic testing. When progression occurred on this agent, a partial disease response was achieved with the combination of nivolumab and ipilimumab.

**Conclusion:** The use of targeted therapies as well as the combination of nivolumab and ipilimumab is a promising option in advanced and recurrent cases of ovarian clear cell carcinoma.

## 1. Introduction

Ovarian cancer affects nearly 20,000 women each year in the United States [1]. Ovarian tumors originate from three cell types: epithelial, stromal, and germ. Most tumors are epithelial, and 10% of epithelial tumors are considered clear cell in histology [2]. Clear cell carcinoma is a Type I tumor, meaning that it often is thought to progress from a precursor lesion [3]. Clear cell carcinoma often presents as a large unilateral pelvic mass and is associated with endometriosis, an elevated venous thromboembolism risk, and hypercalcemia [4]. Many studies have established that earlier stages of clear cell carcinoma have a favorable prognosis, while advanced stages have poorer prognoses than many other ovarian cancers [4, 5]. One study reported that the mean survival time for Stage I/II, Stage III, and Stage IV ovarian clear cell carcinoma is 31.8 months, 12.7 months, and 17.8 months, respectively [4].

Emerging data support the role of immune checkpoint blockade in the management of clear cell ovarian cancer. Specific trials support the use of nivolumab and ipilimumab, which are antiprogrammed cell death 1 (PD-1) and cytotoxic T-lymphocyte-associated Antigen 4 (CTLA-4) therapies, respectively [6, 7]. One study has shown a specific benefit of these therapies in the management of clear cell carcinoma, regardless of PD-1 presence in the tumor [6]. Another study evaluated 20 patients with platinum-resistant ovarian cancer, two of which had clear cell carcinoma. One of the patients with clear cell carcinoma had a complete response to nivolumab for at least 1 year and ongoing at the time of their publication [7].

Here, we present a case of heavily pretreated clear cell carcinoma of the ovary, who had a durable response to nivolumab and ipilimumab.

## 2. Case

**Figure 1:**
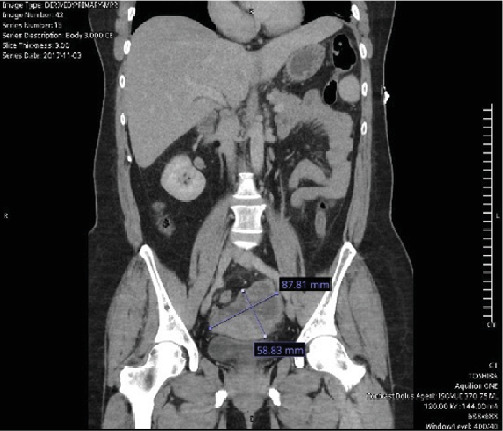
CT scan at time of diagnosis. Coronal image identifying 8.7 *cm* × 5.8 *cm* heterogeneous cystic lesion.

**Figure 2:**
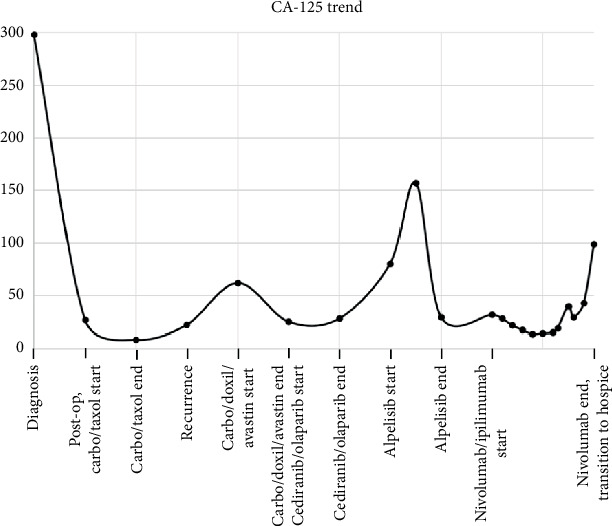
CA-125 trend along treatment course.

A 56-year-old female with a past medical history of hypertension presented to the emergency department (ED) at a large tertiary care center with abdominal pain, chest pain, and nausea in 2017. A computed tomography (CT) scan identified bilateral pelvic masses without signs of metastatic lesions (Figure 1). CA-125 was noted to be elevated at 298 (Figure 2), and a pelvic ultrasound (US) demonstrated large bilateral complex adnexal masses. The right mass measured 3.5 × 2.6 × 2.6 *cm*, and the left mass measured 9.1 × 8.1 × 9.9 *cm*. Gynecologic oncology was consulted. The patient underwent a robot-assisted total laparoscopic hysterectomy, bilateral salpingo-oophorectomy with bilateral ureterolysis, robotic pelvic and aortic lymphadenectomy, peritoneal biopsies, pelvic washings, and mini laparotomy with omentectomy given findings of adenocarcinoma on intraoperative frozen pathology. Evidence of endometriosis was identified during her operation. The final pathology indicated a high-grade clear cell carcinoma with an American Joint Committee on Cancer (AJCC) seventh-edition pathological stage classification of pT2c, pN0, and pMn/a (FIGO Stage IIA). No germline mutations were detected on a Breast and GYN Cancer Panel of 23 genes.

**Figure 3:**
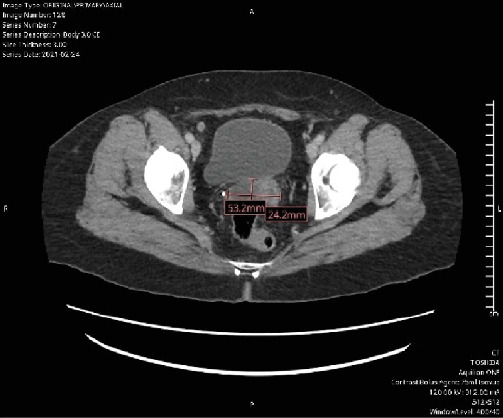
CT scan at time of recurrence. Axial image identifying 5.3 *cm* × 2.4 *cm* complex, low-density cystic lesion.

The patient then received three cycles of carboplatin and paclitaxel and three additional cycles of carboplatin only secondary to persistent Grade 2 neuropathy despite a paclitaxel dose reduction. The patient's CA-125 down-trended throughout her treatment (Figure 2), measuring 9.5 at the completion of her primary chemotherapy, and a CT scan at the end of the six cycles showed no evidence of disease. The patient remained without evidence of disease for 3 years. At that time, the patient went to the ED with right upper quadrant pain. A CT scan indicated a large complex low-density cystic lesion within the pelvis abutting the posterior bladder wall measuring up to 5.1 cm (Figure 3) as well as a subcentimeter nodule at the right hepatic lobe of the liver, overall concerning for recurrent malignancy. A follow-up PET scan supported these findings, additionally identifying a rectus muscle nodule and pelvic nodule. A plan was made for surgical debulking.

The patient underwent an exploratory laparotomy with gynecology oncology including resection of the pelvic mass, abdominal wall mass, and mesenteric mass for recurrent ovarian cancer. The operation identified diffuse disease in the patient's abdomen and pelvis, and optimal reduction was not possible. The final pathology showed metastatic carcinoma, consistent with known ovarian clear cell carcinoma.

The patient received carboplatin and liposomal doxorubicin for two cycles. A CT scan indicated a mixed response to treatment, and the patient had symptomatic improvement. Bevacizumab was added starting Cycle 3. Follow-up CT scan revealed slight regression of disease in the pelvis but with persistent carcinomatosis and a cystic mass in the right pelvic cul-de-sac as well as a new aortocaval necrotic lymph node identified. She was started in a clinical trial where she received cediranib and olaparib with progressive disease after 9 weeks of treatment identified by CT imaging which showed three new liver lesions, a new splenic lesion, and nodules underneath the left hemidiaphragm, concerning for carcinomatosis. Treatment was stopped per study protocol.

RNA and DNA sequence testing identified a gain of function missense variant on Exon 9 of PIK3CA, a frameshift mutation of ARID1A, and a stop gain mutation of ARID1B. Tumor was noted to be microsatellite stable with tumor mutational burden of 58%. PDL1 testing with DAKO PDL1 22C3 clone revealed tumor cell staining (membranous) < 1% and tumor-associated cell staining of 5%.

The patient was started on alpelisib 300 mg daily given PIK3CA mutation [8] via compassionate use program from the drug manufacturer. After 1 month of this treatment, the patient developed hyperglycemia, and the dose of alpelisib was reduced to 250 mg daily. A partial response with a decrease in the size of hepatic and splenic lesions and resolution of ascites was noted. Progression of disease was noted at 14 months, identified by an increasing CA-125 (Figure 2) and CT scan findings.

With consideration of a recent randomized trial [6] showing increased progression-free survival (PFS) of nivolumab and ipilimumab in ovarian clear cell carcinoma, agnostic of PDL1 status, the patient was started on nivolumab 3 mg/kg and ipilimumab 1 mg/kg every 3 weeks for four doses, followed by maintenance nivolumab of 3 mg/kg every 2 weeks. The patient had a partial disease response on this regimen based on both the impression of imaging and the trend of CA-125 (Figure 2), with the best response noted after 12 weeks of induction therapy. She developed pituitary hypophysitis and adrenal insufficiency 5 months after starting therapy. She presented with hyponatremia, fatigue, and failure to thrive. Laboratory studies including ACTH and AM cortisol, LH < FSH, and thyroid studies confirmed the diagnosis. Treatment was with high-dose steroids tapered to hydrocortisone 15 mg in the morning and 5 mg at night. Seven months after starting this regimen, she developed 1.2 cm cerebellar vermis metastases treated with stereotactic radiation. Imaging of the chest, abdomen, and pelvis did not show progressive disease at that time. The patient later developed recurrent adrenal crisis, and a decision was made to enroll in hospice. At that time, imaging showed stable disease in the abdomen, pelvis, and brain. She died 10 months after starting immunotherapy.

## 3. Discussion

Advanced and recurrent ovarian clear cell carcinoma has been shown to have a poor prognosis. One study notes that recurrence is fatal for two out of three patients within 12 months [9]. Another study identified that the mean survival time for Stage II ovarian clear cell carcinoma is 31.8 months and for Stage IV is 17.8 months [4]. Furthermore, studies have identified a short interval between the primary diagnosis of ovarian clear cell carcinoma and its recurrence, with an average of 12.2 months [4]. This data highlights a unique aspect of our patient, with an initial PFS of 3 years and postrecurrence survival of 35 months.

Epithelial ovarian cancers, including clear cell carcinoma, are treated with platinum-based chemotherapy regimens as the first line; however, clear cell carcinoma has been shown to have a poorer response to these regimens compared to serous carcinomas [4]. Multiple clinical trials explore alternative chemotherapy regimens in the treatment of clear cell given this suboptimal response [10, 11].

Immune checkpoint modulators, specifically anti-CTLA-4 and anti-PD-1 therapies, are an area of interest. One way tumor cells evade the immune system is through CTLA-4 and PD-1 pathways; thus, these agents ultimately work by inhibiting the tumor cell's ability for evasion [12]. Studies evaluating checkpoint inhibitor treatment of high-grade epithelial ovarian cancers have been unsuccessful [13–15]. However, clear cell carcinoma has shown a more durable response than other epithelial ovarian cancers to checkpoint therapies, likely due to related differences in tumor biology such as higher rates of microsatellite instability, tumor infiltrating lymphocytes, and PDL1 expression [7, 16, 17].

Recent research studies evaluate the use of nivolumab in the treatment of ovarian cancers [7]. However, larger trials showed a modest response to anti-PD-1 agents alone [18], and the addition of anti-CTLA-4 was proven to be more efficacious in multiple studies [19–21]. A recent randomized control trial explored the efficacy of nivolumab versus nivolumab plus ipilimumab for recurrent or persistent ovarian cancer. The data demonstrated a superior response rate to nivolumab plus ipilimumab versus nivolumab alone, including a greater response rate at 6 months of treatment and improved PFS, with similar reported toxicities. Patients with clear cell ovarian cancer appeared to benefit the most from the combination; however, only 12 patients in the study had clear cell histology [6].

Pembrolizumab is also reported in the setting of heavily pretreated patients with ovarian clear cell carcinoma. A case series reported a median of 12.2 weeks of PFS and a median overall survival of 71.0 weeks [22]. That same study identified common side effects as hyperthyroidism, acute kidney injury, elevated liver function tests, anemia, encephalitis, and diabetic ketoacidosis [22]. There are no studies directly comparing pembrolizumab and the combination of nivolumab and ipilimumab in the treatment of ovarian clear cell carcinoma.

Interestingly, this patient also showed a durable response to alpelisib. The PTEN-PI3K-AKT pathway is frequently altered in gynecological tumors [23]. The medication is FDA approved for treatment of PIK3CA-mutated advanced breast cancer in combination with fulvestrant [24]. Data regarding the use of alpelisib in gynecologic cancers is lacking, although preclinical data suggests its benefit in the treatment of ovarian clear cell carcinoma [25]. Clinically, a Phase Ib study evaluating treatment with alpelisib and olaparib in germline nonmutated BRCA (gBRCAwt) recurrent ovarian cancer showed an objective response rate (ORR) of 31% which is favorable to the ORR for single-agent olaparib in BRCA-negative or unknown cancers [26]. A Phase III trial is ongoing to evaluate the combination. A recent case report supports the use of alpelisib, demonstrating a patient with a PIK3A-mutated mixed endometrioid serous and clear cell ovarian cancer in complete remission for 2 years following its use [27]. Further, a recent case series supports the use of alpelisib in PIK3A-mutated advanced gynecologic cancers, showing an ORR of 28% and disease control rates (DCRs) of 61% [28].

This case supports the use of targeted therapy and next-generation sequencing in the treatment of clear cell ovarian cancer. After progression on platinum-based chemotherapy and a clinical trial, the patient had a durable response to alpelisib. Following progression on this agent, a partial response with 7-month progression-free survival is observed with the combination of nivolumab and ipilimumab. This patient's response to nivolumab and ipilimumab surpasses that supported in current data. A recent study showed PFS of 3.9 months with nivolumab plus ipilimumab when used in patients with platinum-resistant ovarian cancer [6]. More so, a publication identifies the median time to treatment failure with these therapies to be 99 days [29].

This case emphasizes the importance of close patient follow-up and adjustments to therapeutic regimens. It serves as an example of successful treatment of advanced recurrent ovarian clear cell carcinoma and supports the continued study of targeted treatments in the historically treatment-refractory ovarian cancer histology.

## Data Availability

Data sharing is not applicable to this article as no new data were created or analyzed in this study.
